# High Density Lipoprotein Cholesterol Increasing Therapy: The Unmet Cardiovascular Need

**Published:** 2014-09-01

**Authors:** Giovanni Cimmino, Giovanni Ciccarelli, Alberto Morello, Michele Ciccarelli, Paolo Golino

**Affiliations:** 1 Department of Cardiothoracic and Respiratory Sciences, Second University of Naples, Italy;; 2 Department of Medicine, University of Salerno, Italy

**Keywords:** Atherosclerosis, Reverse Cholesterol Transport, Cholesterol Efflux, Apolipoprotein AI, HDL

## Abstract

Despite aggressive strategies are now available to reduce LDL-cholesterol, the risk of cardiovascular events in patients with coronary artery disease remains substantial. Several preclinical and clinical studies have shown that drug therapy ultimately leads to a regression of the angiographic lesions but also results in a reduction in cardiovascular events. The dramatic failure of clinical trials evaluating the cholesterol ester transfer protein (CEPT) inhibitors, torcetrapib and dalcetrapib, has led to considerable doubt about the value of the current strategy to raise high-density lipoprotein cholesterol (HDL-C) as a treatment for cardiovascular disease. These clinical results, as well as animal studies, have revealed the complexity of HDL metabolism, assessing a more important role of functional quality compared to circulating quantity of HDL. As a result, HDL-based therapeutic interventions that maintain or enhance HDL functionality, such as improving its main property, the reverse cholesterol transport, require closer investigation. In this review, we will discuss HDL metabolism and function, clinical-trial data available for HDL-raising agents, and potential strategies for future HDL-based therapies.

## I. INTRODUCTION

In the last ten years, several therapeutic interventions have been developed to reduce Low Density Lipoprotein-Cholesterol (LDL-C) to meet the gold standard levels as suggest by the current guidelines [1]. However, the cardiovascular risk remains still significant, approaching an annual risk of 9% in patients with established coronary artery disease (CAD), thus novel strategies are needed for targeting this residual cardiovascular risk [2]. In this regard, early epidemiological studies have identified low levels of high-density lipoprotein cholesterol (HDL-C, <1.0 mmol/l or 40 mg/dl) to be an independent risk factor for cardiovascular disease [3]. Therefore, raising HDL-C levels provides an important strategy for addressing the residual cardiovascular risk in these patients. Experimental studies have identified several beneficial functions of HDL that seems to be independent of its primary cholesterol removal. They include endothelial [4, 5], antiapoptotic [6], antithrombotic [7], antioxidant [7], and anti-inflammatory effects [8], all of which may contribute to its anti-atherosclerotic properties. Several important lifestyle changes, such as aerobic exercise, smoking cessation, weight loss reduction, “low-fat” diet and high intake of n-3 polyunsaturated fats, mild to moderate alcohol intake, etc. can help to raise HDL-C levels [9]. Unfortunately, these strategies are often not enough to reach the suggested HDL-C levels. Current pharmacological approaches include nicotinic acid, fibrates, statins, thiazolidinediones, bile acid sequestrants in high doses have been employed, but the side effects and the high variability in raising HDL, significantly hinder their wide use [3, 9]. Furthermore, the disappointing results of the ILLUMINATE trial with the cholesteryl ester transfer protein (CETP) inhibitor, torcetrapib [10], the early termination of dal-OUTCOMES with dalcetrapib because of no benefits [11], strongly suggest that the “quality” of HDL is also extremely important and novel and more effective strategies are needed to adequately increase HDL-C. Moreover, a recent meta-analysis [12] has shown that niacin, CEPT inhibitors and fibrates, targeted at increasing HDL-C levels, were not associated with significantly reduced risk of all cause mortality and coronary heart disease mortality.

The major difference between the LDL and HDL is their protein moiety. Apolipoprotein A-I (ApoA-I) is the major protein constituent of HDL [13], defining its size and shape, solubilizing its lipid components, removing cholesterol from peripheral cells, activating the LCAT enzyme, and delivering the resulting cholesterol esters to the liver, a phenomenon called reverse cholesterol transport (RCT). Raising ApoA-I levels and then nascent HDL, seems to be one of the most promising strategy [13, 14]. Several approaches have been attempted in recent years to increase HDL-C levels, including the direct administration of synthetic HDL [15] or ApoA-I, as a whole [16] or as a mimetic peptide [17]. Furthermore, pre-clinical [18] and clinical [19] studies have previously shown that intravascular infusions of synthetic HDL containing a genetic variant of ApoA-I, ApoA-I Milano (ApoA-I_M_), inhibit progression and induce rapid regression and remodelling of atherosclerosis. Also ApoA-I gene transfection results in sustained expression of functional ApoA-I and increased plasma HDL cholesterol [20]. More important, this increase is associated with plaque regression and stabilisation [21].

## II. REVERSE CHOLESTEROL TRANSPORT AND HIGH-DENSITY LIPOPROTEIN CHOLESTEROL FUNCTIONS

**FIGURE 1:**
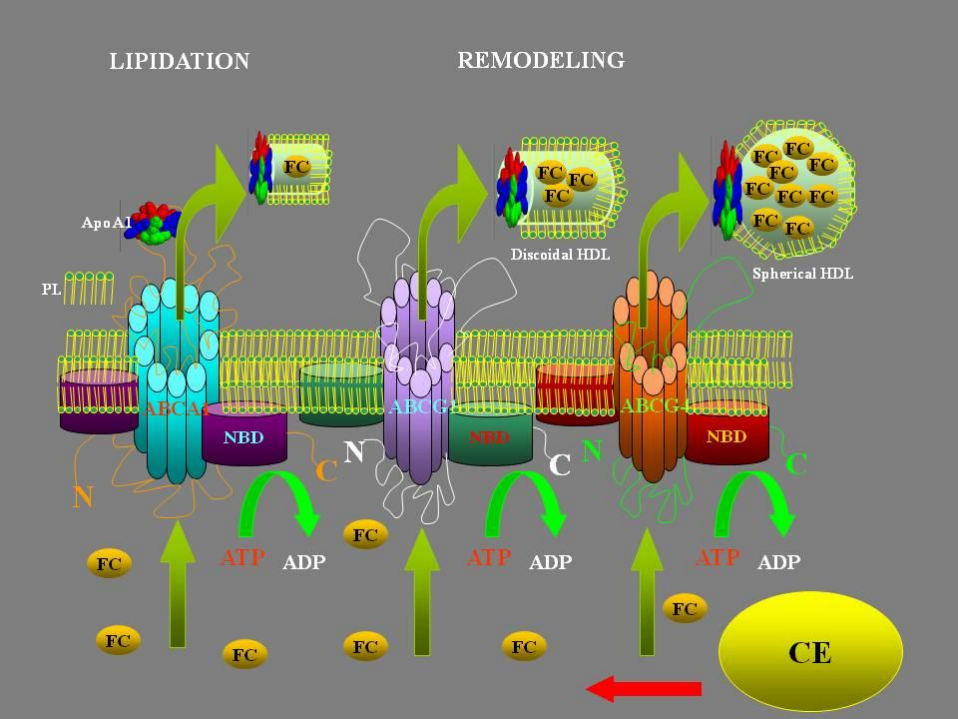
*HDL formation:* HDL particles start out as apolipoproteins produced by the liver, called apoAI. Precursor molecules are released in HDL called pre-B-HDL, incorporating small quantities of cholesterol and lipids, especially phospholipids (PL). ABC proteins (ATP-Binging Cassette Transports) transport various molecules across extra- and intra-cellular membranes. Cholesterol from non-hepatic peripheral tissues is transferred to HDL by the ABCA1. ABCG1 and ABCG4 are necessary for the further lipidation. These receptors are required for spherical particles HDL formation. The free cholesterol (FC) is converted to cholesteryl esters (CE) by the enzyme LCAT (lecithin-cholesterol acyltransferase).

Although the atheroprotective properties of HDL-C have not been directly compared, it is likely that RCT plays a crucial role in these anti-atherogenic effects [22]. The RCT hypothesis proposes that HDL-C accepts cholesterol from the periphery, such as arterial wall cells, and deliver it to the liver for excretion via the bile salts. In 1990 the pioneering work of Badimon et al. [23] employed HDL-C as a therapeutic agent: it was the first preclinical evidence that plaque regression is feasible, showing that HDL-C infusion promotes regression of pre-existing lesions in a rabbit model of atherosclerosis. Other HDL-C elevating interventions corroborated this finding in animals [18] and in humans [15, 19]. The life cycle of HDL begins with apolipoprotein A-I (ApoA-I) being synthesized by the liver and, after interaction with hepatic ATP-binding cassette transporter 1 (ABCA1), is secreted into plasma as lipid-poor ApoA-I [14, 22]. The maturation process starts by acquisition of cholesterol and phospholipids (PLs) via ABCA1-mediated efflux from the liver and the transfer of cholesterol, PLs, and apolipoproteins from chylomicrons and very low-density lipoproteins (VLDL) during lipoprotein-lipase-mediated lipolysis to form nascent pre-β-HDLs [13]. Additional cholesterol and PLs are acquired from cells in extrahepatic tissues via ABCA1-mediated efflux, progressively generating more cholesterol-enriched particles. The enzyme lecithin-cholesterol acyl transferase (LCAT), carried on HDLs, esterifies the free cholesterol (FC) to cholesteryl ester (CE), which migrate to the core of the HDL-particle to form mature HDLs that can acquire additional lipid via ABCG1 and SR-BI-mediated efflux [13, 14, 22] (Figure 1). Thus, pharmacological modulation of any of the key players in RCT may be of great importance for cholesterol accumulation and/or removal, ultimately modifying the atherosclerotic process.

## III. NIACIN, CEPT INHIBITORS AND FIBRATES: IT HAS BEEN ONLY AN ILLUSION!

### Niacin

Niacin, also known as vitamin B3 and nicotinic acid, is a physiological precursor of two coenzymes (nicotinamide adenine dinucleotide and nicotinamide adenine dinucleotide phosphate) involved in oxidoreductive reactions and energy metabolism [24]. The chronic deficiency of niacin leads to the onset of Pellagra, a disease characterized by dementia, dermatitis, and diarrhea [25]. Niacin reduces all proatherogenic lipid and lipoprotein particles, including total cholesterol, triglycerides, VLDL, LDL, Lp (a) and small dense LDL particles [26, 27] and it is, therefore, the most potent available drug to increase antiatherogenic HDL levels [27]. Moreover, niacin increases larger HDL2 subfractions and selectively increases apoA-I-containing HDL particles, a mediator of RCT [28–30]. There are several pathways related to increase of HDL in patients treated with niacin. By inhibiting the surface expression of hepatocyte β chain ATP synthase [31] (a recently recognized HDL holoparticle receptor), niacin decreases the uptake of HDL-apoAI without affecting the de-novo synthesis of apoAI in Hep-G2 cells [32, 33]. Niacin, therefore, could down-regulates cell surface expression of the β chain ATP synthase, leading to hepatic removal of HDL through holoparticle endocytosis, retaining increased HDL-apoAI particles in the bloodstream. At the same time, niacin increases apoA-I lipidation and HDL biogenesis by enhancing lipid efflux through a DR4-mediated transcription of ABCA1 gene in hepatocytes [27, 34]. Regarding the effect on triglycerides, niacin inhibited hepatocyte triglyceride synthesis and increased intracellular post-translational apoB degradation resulting in decreased secretion of apoB and apoB-containing VLDL and LDL particles [35]. In particular, niacin directly and non-competitively inhibited hepatocyte microsomal DGAT2, a key enzyme that catalyzes the final reaction in triglyceride synthesis as the target site for niacin’s action on triglyceride synthesis [36]. In this way, niacin, by decreasing atherogenic VLDL/LDL particles and increasing antiatherogenic HDL, contribute to decrease atherosclerosis. Moreover, niacin seems to exert also pleiotropic effects. It has been recently reported that niacin significantly inhibited reactive oxygen species (ROS) production, LDL oxidation, redox-sensitive vascular cell adhesion molecule-1 (VCAM-1) and monocyte chemotactic protein-1 (MCP-1) mRNA expression, and monocyte adhesion in human aortic endothelial cells [26], suggesting that this drug inhibits vascular inflammation by decreasing endothelial ROS production, and subsequent expression of critical inflammatory VCAM-1 and MCP-1 genes, that have an important role in the development of atherogenesis. In addition to the vascular effects, recent studies have also shown that niacin inhibits atherosclerosis in mice via its receptor GPR109A expression in macrophages and immune cells [37, 38].

The most important data to sustain the use of niacin derive from the Coronary Drug Project (CDP), a pre-statin study evaluating niacin monotherapy in patients with prior myocardial infarction. Niacin was associated with a 27% reduction in the incidence of nonfatal re-infarction at 6 years [39], and all-cause mortality was reduced by 11% at 15 years [40]. Different pharmaceutical formulations have been created to avoid the adverse effects, in particular flushing, with the addition of prostaglandins inhibitors. Its therapeutic use has been considered for decades in the prevention and treatment of atherosclerosis but, in the last years, negative outcomes of recent clinical trials, have questioned its effectiveness [41]. Two recent studies have evaluated the role of niacin in the reduction of cardiovascular risk. The results of AIM-HIGH trial (Atherothrombosis Intervention in Metabolic Syndrome With Low HDL/High Triglycerides: Impact on Global Health Outcomes) showed, in an interim analysis, no cardiovascular benefit from taking extended-release niacin when added to therapy with a simvastatin plus ezetimibe. In fact, there was a greater risk of ischemic stroke, which did not reach statistical significance with a early discontinuation of the study [42]. HPS2-THRIVE trial, instead, which evaluated the CordaptiveTM/statin combination versus statin alone, was recently stopped after only 3.9 years because of serious adverse events in the treated arm [43]. On the other hand, a recent meta-analysis of 11 clinical trials, including AIM-HIGH, in about 10000 patients, showed that niacin therapy was associated with a significant reduction in major Cardiovascular disease and coronary heart disease events [44]. Accordingly, there is a need for further studies designed to evaluate the role of niacin in combination with the new cholesterol-lowering drugs in reducing the incidence of CVD events.

### Cholesteryl ester transfer protein inhibitors

Considering the role of CETP in RCT, which favors transfer of cholesterol from HDL to LDL, it was thought that a pharmacological action against this enzyme could reduce the risk of atherosclerosis, and synthetic inhibitors were developed to restore a favourable lipoprotein profile by increasing HDL-C concentration and lowering LDL-C concentration. Torcetrapib was the first molecule to be designed as CETP inhibitor, and evaluated in phase III of ILLUMINATE trial [45]. The study was stopped due to a global overall mortality in patients treated with this drug, although HDL-C concentration had increased. Subsequent analyses showed that this negative effect was due to activation of the renin-angiotensin-aldosterone system, increasing blood pressure, but also to direct vascular endothelium toxicity [46]. Despite the failure of ILLUMINATE trial, new drugs were synthesized: dalcetrapib, evacetrapib, and anacetrapib. Dalcetrapib is a CETP inhibitor that raised HDL cholesterol levels by approximately 30%, without significant effects on LDL cholesterol levels, blood pressure, or circulating neurohormones but with possible beneficial vascular effects, including the reduction in total vessel enlargement over 24 months [47, 48]. In dal-OUTCOMES study, were evaluated the effects of dalcetrapib on cardiovascular risk among patients with a recent acute coronary syndrome. The addition of dalcetrapib to standard therapy after an acute coronary syndrome raised the levels of HDL cholesterol and apo-AI, with minimal effects on levels of LDL cholesterol and apo-B. Nevertheless, the risk of major cardiovascular outcomes was not significantly altered [11]. As regards the other two drugs, are still ongoing trials to demonstrate their effectiveness. The safety of evacetrapib has been demonstrated in a phase II study [49], to evaluate its efficacy and safety versus placebo in high-risk patients for vascular disease will end in January 2016. Two phase III trials are currently ongoing for anacetrapib: the DEFINE trial [50] and the REVEAL HSP-3 TIMI-55 trial (Randomized Evaluation of the Effects of Anacetrapib through Lipid Modification). In addition, a recent paper shown that after the withdrawal of active treatment, anacetrapib plasma lipid changes and drug levels decreased to approximately 40% of on-treatment trough levels at 12 weeks after dosing, but modest HDL cholesterol elevations and low drug concentrations were still detectable 2 to 4 years after the last dosing [51]. In the DEFINE trial, conducted in patients at high risk for coronary heart disease, anacetrapib reduced LDL cholesterol levels by 39.8% after 24 weeks compared with placebo, and demonstrated an acceptable safety profile. Furthermore, a raise of 138.1% in HDL cholesterol levels, with no alterations in blood pressure, aldosterone or electrolytes has been reported. The REVEAL HSP-3 TIMI-55 trial, which includes 30,000 coronary patients treated with statins, will end in 2017. Inhibiting CETP could have an anti-atherogenic effect because it inhibits the movement of cholesteryl esters into apo-B-containing lipoproteins with a decreasing of the cholesteryl esters-laden LDL available for uptake by macrophages for foam cell formation. However, inhibiting this HDL ‘rearrangement”, may diminish apoA-I and lipid-poor HDL pools available for peripheral cholesterol efflux or may decrease the possibility of cholesteryl esters being transferred to apo-B-containing lipoproteins for excretion by the liver, thus defining a pro-atherogenic effect. Furthermore, it is known that there are different subtypes of LDL that can be separated in two phenotypes differing, in particular, in size, density, and atherogenicity. These phenotypes have been called ‘pattern A’ (or larger LDL) and ‘pattern B’ (or smaller LDL) [52]. LDL size correlates positively with plasma HDL levels [53] and several studies have demonstrated that the phenotype B is correlated with an increased cardiovascular risk [52]. Therefore, through inhibition of CETP, could increase the level of LDL smaller that would increase the risk of atherosclerosis.

### Fibric acid derivatives

Peroxisome proliferator-activated receptors (PPARs) are nuclear transcription receptors that regulate lipid and carbohydrate homeostasis; three isoforms (α, γ and ß/δ) are known. Fibric acid derivatives or fibrates, are agonists of α isoform of PPARs. They have several functions on lipids levels in bloodstream, among others: increase HDL-C levels (by 10%–20%), modestly lower LDL-C levels (by 10%–15%), and substantially lower levels of triglycerides (by 40%–50%) [54, 55]. Moreover, these drugs improve lipoprotein plasma profile and insulin sensitivity while enhance vasomotor reactivity, and reduce inflammation [56]. Several clinical studies have assessed the impact of fibrates on clinical outcomes in primary and secondary prevention (the Helsinki Heart Study, the VA-HIT study, etc). Despite a 34% reduction in major coronary events at 5 years, as shown by the Helsinki Heart Study, fibrates have failed to reduce overall mortality in the general population [12]. In the VA-HIT trial, treatment with gemfibrozil was associated with reduction in CVD events. The study showed a reduction in the combined outcome of cardiovascular death, non-fatal myocardial infarction, and stroke of 24 percent, attributed to a modest increase (6%) in HDL-C levels [55]. Some evidence indicate that the benefits seen in the VA-HIT cohort may be partly due to additional effects of fibrates on apolipoprotein synthesis and lipoprotein metabolism [57]. The combination of fibrates, particularly gemfibrozil, with statins requires caution and monitoring of creatine kinase levels because of the risk for myotoxicity, including rhabdomyolysis [58]. Because of variable-outcome study results and problems linked to the safety, the precise role of fibrate treatment remains uncertain but is reasonable that fenofibrate and bezafibrate could be useful for diabetes or metabolic syndrome and gemfibrozil could be useful for patients with dyslipidemia [58, 59]. A new class of drugs, called glitazars, are dual agonists acting on both α and γ isoforms and are able to impact lipid and carbohydrate metabolism. Positive effects on lipid metabolism and insulin-sensitizing make possible their use in metabolic syndrome in patients with low HDL. In the phase II of SYNCHRONY study [60] was evaluated, in type II diabetics patients, the safety and positive impact on lipoprotein profile of aleglitazar. A phase III study (AleCardio), including patients with type 2 diabetes hospitalized for acute coronary syndrome, was halted for increase in incidence of bone fractures, heart failure, and gastrointestinal bleeding.

## IV. RECOMBINANT HDL INFUSION AND APO-AI INCREASING STRATEGIES: A LOOK TO A CLOSE FUTURE?

In 1990, the pioneering study of Badimon et al. employed HDL as therapeutic agent. It was the first preclinical evidence that plaque regression is feasible, showing that HDL cholesterol infusion regressed preexisting lesions in a rabbit model of atherosclerosis [23]. Other LDL-lowering interventions [61, 62] corroborated this finding in different models [4, 7, 8, 63], while several HDL-elevating interventions have reduced atheroma volume in humans [14]. Intravenous administration of liposomic complexes containing human proapolipoprotein A-I (the secreted form of apoA-I) in patients with familial hypercholesterolaemia, led a raise of 30% of fecal excretion of cholesterol and biliary acids [64]. Purified apoA-I was then combined with soya phophatidylcholines and the product was called CSL-111 that was tested in patients with acute coronary syndrome [15]. After a weekly perfusion over 1 month, patients treated with CSL-111 had a 3.4% volume reduction in the atherosclerotic plaque but was associated with mild, self-limiting transaminase elevation, clinically well tolerated. Recently, second generation products have emerged, such as CSL-112 and CER-001, currently evaluated in ongoing studies. In the presence of human plasma, CSL-112 was significantly more potent than native HDL at enhancing cholesterol efflux from macrophages, and the efflux elevation was predominantly via the ABCA1 transporter [65]. Phase 2 trials of CSL-112 are currently under way in stable coronary artery disease and ACS patients. At the same time, administration of CER-001 [66, 67] caused elevations in plasma cholesterol, as well as, total and free cholesterol in HDL fraction suggesting increased reverse cholesterol transport. In the 80s, in a Town of Northern Italy (Limone sul Garda), in three members of a family with significant hypertriglyceridaemia and low HDL-C concentrations, without any manifestation of atherosclerosis, was discovered a new type of apoA-I, called apoA-I Milano (apoA-I_M_). It is a natural variant of apoA-I characterized by a cysteine for arginine substitution at position 173 of the primary sequence. ApoA-I_M_ carriers have much less atherosclerosis than expected from their very low HDL cholesterol levels, suggesting that the variant might be protective [18]. Despite a similar effect on acute plaque regression, the infusion of HDL-Milano exerts superior anti-inflammatory and plaque stabilizing effects than HDL wild-type in the short term [68]. Synthetic HDLs, made with a recombinant form of apoA-I_M_ and phospholipids given in two injections four days apart, are effective in inducing the regression of atherosclerotic plaques, in enhancing reverse cholesterol transport [69], in reducing global inflammation [69, 70], in preventing arterial restenosis, limiting cardiac dysfunction after ischemia/reperfusion injury [18, 19], and even in reversing aortic stenosis [71].

### ApoA-I mimetic peptide

Recently, novel HDL-directed target therapies have been created. FAMP (Fukuoka University ApoA-I Mimetic Peptide) is a drug that mimics human apoA-I without complexing with Phospholipids. It enhances the function of HDL, and suppresses aortic plaque formation in apoE-knockout mice fed a high-fat diet [72]. FAMP markedly increases pre-β HDL and overall cholesterol efflux from peripheral tissues. It may enhance cellular cholesterol efflux trough internalization and transcytosis in macrophages or aortic endothelial cells, and the removal of intracellular cholesterol. Therefore, is described a physical interactions between FAMP and ABCA1 to transport intracellular cholesterol to the circulation, and this latter mechanism may be predominant. Another apoA-I mimetic peptide, 4F, removes plasma oxidized lipids (PLs and fatty acids) and improves the anti-inflammatory properties of HDL [73]. Because of the very high cost of the latter, have been developed transgenic tomato plants expressing an apoA-I mimetic peptide, 6F. After 13 weeks of treatment with this drug, there was a reduction in the percent of aorta with lesions than the control group [17].

### Apo AI gene therapy

The biggest limitations in the use of HDL are the costs and the inability to maintain these stable levels of HDL after injection. In a previous study we have compared the safety and effectiveness of a single administration of AAV8-based vectors encoding human ApoA-I, driven by cytomegalovirus promoter, after intraportal and intramuscular injection over a period of 16 weeks [20]. Hepatic and muscular gene transfers of human ApoA-I in ApoA-I–null mice using AAV8 vectors resulted in sustained expression of functional ApoA-I and increased plasma HDL cholesterol. These data suggest that intramuscular AAV8-mediated gene transfer of human ApoA-I is as effective as intraportal vector delivery in increasing circulating ApoA-I and HDL-cholesterol levels. As such, intramuscular gene delivery of ApoA-I may serve as an appealing minimally invasive technique in the management of atherosclerosis. Furthermore, these strategies resulted also into a significant reduced progression of established atherosclerotic lesions in an animal model of atherosclerosis [74]. Therefore, de novo ApoA-I production is an important driving force behind HDL elevation and gene transfer by viral vector seems a promising strategy to pursue.

## V. RATIONALE TO INCREASE APO-AI

Clinical and basic research has proven that not all HDL particles possess antiatherogenic properties since chronic inflammatory processes render HDL particles dysfunctional [75, 76] with alterations of their structure and metabolism. Some of the already used pharmacological agents do not increase the “protective” HDL. From the physiological point of view, raising ApoA-I should provide the way to increase nascent empty HDL [73]. Preclinical studies [18, 71, 74] strengthen this hypothesis.

## VI. CONCLUSIONS

Current guidelines on lipid management focus on reducing the amount of circulating LDL cholesterol through the body because of the availability of powerful LDL-lowering medications, namely the statins, which are ideal agents for long-term use and have proven their effectiveness in large randomized clinical trials. However, it is also widely accepted that other aspects of lipid metabolism could produce important therapeutic targets to control the epidemic of atherosclerosis and consequent cardiovascular disease. Interventions on the HDL side of a patient’s lipid profile have been typically based on the concept that raising HDL cholesterol levels will reduces atherosclerosis. This expectation comes from epidemiological observations consistently showing an inverse correlation between HDL cholesterol levels and cardiovascular event rates (i.e., myocardial infarctions). Nonetheless, direct evidence that raising HDL cholesterol levels will reduce cardiovascular risk is scarce and incomplete. A suggestive hypothesis could be that low levels of HDL-C did not constitute a risk factor *per se*, but only an indirect marker of cardiovascular risk, as they are often associated with obesity, impaired glucose tolerance and diabetes. This could be the reason for the absence of a clear efficacy of the drugs tested to date, as the therapeutic target does not appear to be the correct one. In particular, despite of high hopes on the use of CETP inhibitors, the occurrence of side effects linked to the molecule and, at the same time, to the pharmacologic class, advises against the use of such drugs. The failure of the trials maybe related to a “clogging” of HDL, which would no longer be able to effectually carry out their amount of cholesterol. To date, then, the real effective treatment lowering HDL-C remains the non-pharmacological therapy, represented by regular physical activity. Furthermore, current strategies still fail to increase HDL cholesterol, thus novel and more promising intervention, such as direct injection of “good quality” HDL or over-expression of ApoA-I, are needed to reach the gold standard therapy in CVD management.

## Figures and Tables

**FIGURE 2:**
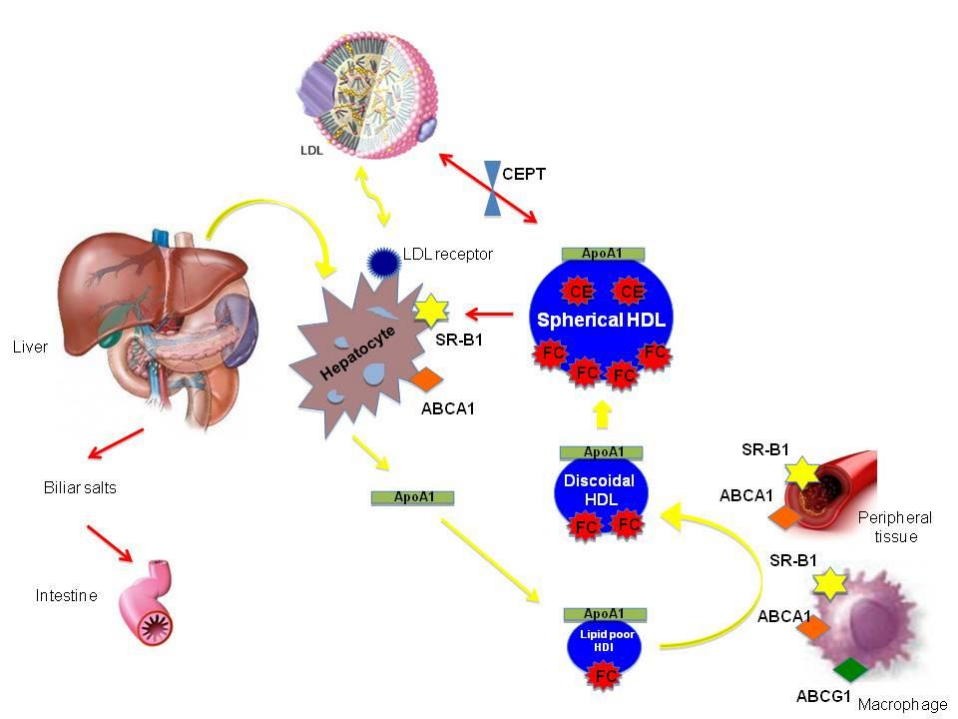
*Schematic view of RCT:* ApoA-I is responsible for the metabolic fate of HDL cholesterol HDLs and is synthesized by the liver. HDLs are released to the circulation as a lipid-poor HDL (nascent HDL), mostly formed by ApoA-I and phospholipids (PL). Through their metabolic maturation, HDLs interact with the ABCA1, ABCG1 and also SR-B1 receptor in the macrophage surface and peripheral tissue increasing their lipid content by taking PLs and cholesterol becoming spherical HDL. The cholesterol of the HDLs is transported to the liver, via the scavenger receptor class B, type I (SR-BI), for further metabolization and excretion to the intestines in the form of bile acids and cholesterol, completing the process of reverse cholesterol transport.

**FIGURE 3:**
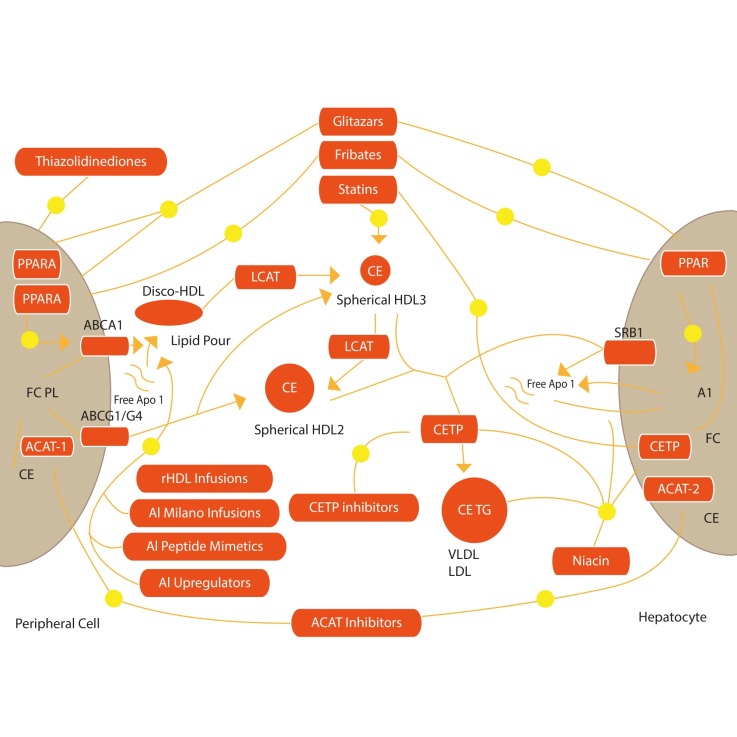
*Therapeutic Interventions to increase RCT:* This is a schematic view of the possible strategies to increase Apo AI functions, via Apo AI mimetic peptides, ApoA-I Milano infusions, ApoA-I viral transfection, recombinant HDL infusion. These interventions may result in an increased RCT, thus removing cholesterol from the body and reducing atherosclerosis
